# Supplementary material for: Splice-Junction-Based Mapping of Alternative Isoforms in the Human Proteome
Source: Cell Rep. Author manuscript; Available in PMC 2020 Jan 15. (PMC6961840; doi:10.1016/j.celrep.2019.11.026)
Supplement: 2 [file NIHMS1546469-supplement-2.zip › DF1/PXD010154/Prostate/Prostate_6_CHTF18_QVDADLQPAGSLPHGASTPSPSGVPLFTAG.pdf]

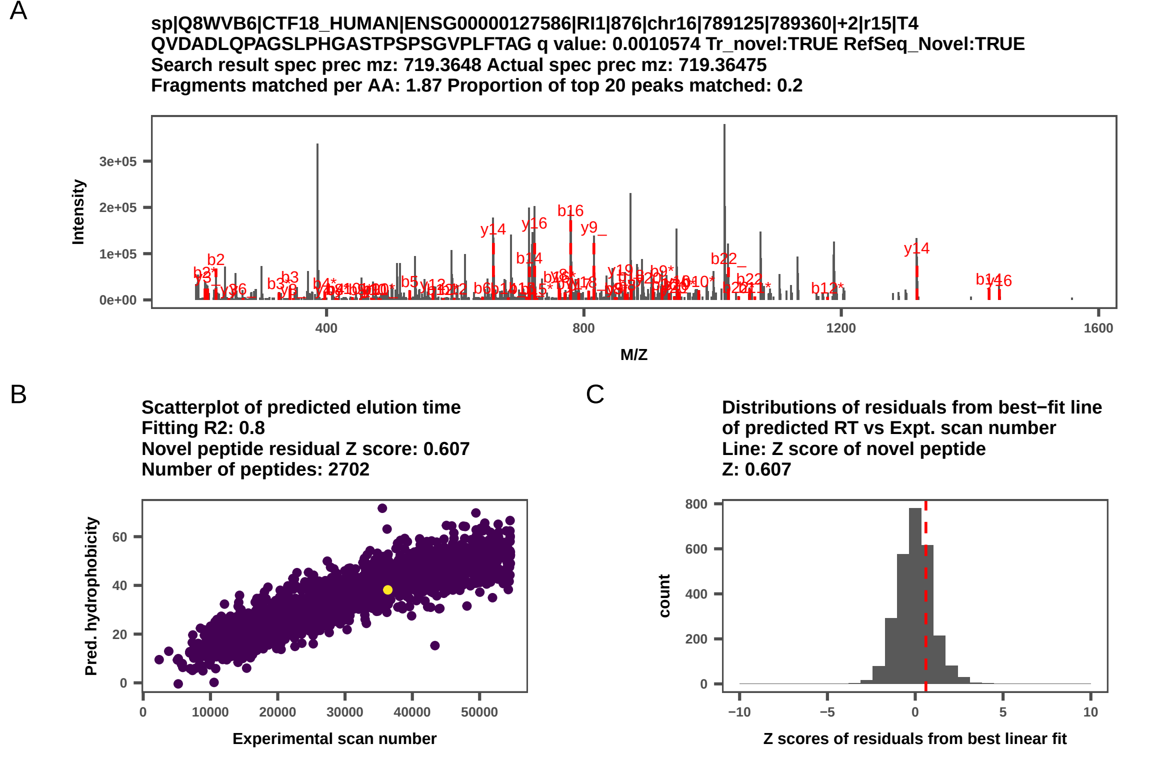
A
sp|Q8WVB6|CTF18_HUMAN|ENSG00000127586|RI1|876|chr16|789125|789360|+2|r15|T4
QVDADLQPAGSLPHGASTPSPSGVPLFTAG q value: 0.0010574 Tr_novel:TRUE RefSeq_Novel:TRUE
Search result spec prec mz: 719.3648 Actual spec prec mz: 719.36475
Fragments matched per AA: 1.87 Proportion of top 20 peaks matched: 0.2
Intensity
3e+05
2e+05
1e+05
0e+00
y14
y16
b16
y9_
b14
b2
b22_
y14
b12*
400
800
1200
1600
M/Z
B
Scatterplot of predicted elution time
Fitting R2: 0.8
Novel peptide residual Z score: 0.607
Number of peptides: 2702
Pred. hydrophobicity
60
40
20
0
0
10000
20000
30000
40000
50000
Experimental scan number
C
Distributions of residuals from best−fit line
of predicted RT vs Expt. scan number
Line: Z score of novel peptide
Z: 0.607
count
800
600
400
200
0
−10
−5
0
5
10
Z scores of residuals from best linear fit
